# An ant colony optimization algorithm for phylogenetic estimation under the minimum evolution principle

**DOI:** 10.1186/1471-2148-7-228

**Published:** 2007-11-15

**Authors:** Daniele Catanzaro, Rafflaele Pesenti, Michel C Milinkovitch

**Affiliations:** 1 Laboratory of Evolutionary Genetics, Institute for Molecular Biology and Medicine (IBMM), Université Libre de Bruxelles (U.L.B.), CP300, Rue Jeener et Brachet 12, B-6041, Gosselies, Belgium; 2 Dipartimento di Matematica Applicata, Universitá Ca' Foscari, Dorsoduro 3246 - 30123, Venice, Italy

## Abstract

**Background:**

Distance matrix methods constitute a major family of phylogenetic estimation methods, and the minimum evolution (ME) principle (aiming at recovering the phylogeny with shortest length) is one of the most commonly used optimality criteria for estimating phylogenetic trees. The major difficulty for its application is that the number of possible phylogenies grows exponentially with the number of taxa analyzed and the minimum evolution principle is known to belong to the NP
 MathType@MTEF@5@5@+=feaafiart1ev1aaatCvAUfKttLearuWrP9MDH5MBPbIqV92AaeXatLxBI9gBaebbnrfifHhDYfgasaacPC6xNi=xH8viVGI8Gi=hEeeu0xXdbba9frFj0xb9qqpG0dXdb9aspeI8k8fiI+fsY=rqGqVepae9pg0db9vqaiVgFr0xfr=xfr=xc9adbaqaaeGacaGaaiaabeqaaeqabiWaaaGcbaWenfgDOvwBHrxAJfwnHbqeg0uy0HwzTfgDPnwy1aaceaGae8xdX7Kaeeiuaafaaa@3888@-hard class of problems.

**Results:**

In this paper, we introduce an Ant Colony Optimization (ACO) algorithm to estimate phylogenies under the minimum evolution principle. ACO is an optimization technique inspired from the foraging behavior of real ant colonies. This behavior is exploited in artificial ant colonies for the search of approximate solutions to discrete optimization problems.

**Conclusion:**

We show that the ACO algorithm is potentially competitive in comparison with state-of-the-art algorithms for the minimum evolution principle. This is the first application of an ACO algorithm to the phylogenetic estimation problem.

## Background

The Minimum Evolution (ME) principle is a commonly used principle to estimate phylogenetic trees of a set Γ of *n *species (taxa) given an *n *× *n *symmetric matrix **D **= {*d*_*ij*_} of evolutionary distances. First introduced by Kidd and Sgaramella-Zonta [1] and subsequently reinterpreted by Rzhetsky and Nei [2,3], the ME principle aims at finding a phylogeny characterized by minimal sum of branch lengths, under the auxiliary criteria that branches have a positive length and the pair-wise distances on the tree are not smaller than the directly observed pair-wise differences. Its biological justification is based on the fact that, when unbiased estimates of the true distances are available, the correct phylogenetic tree has an expected length shorter than any other possible tree [2,3] compatible with the distances in **D**. More formally, the ME principle can be expressed in terms of the following optimization problem:

**Problem 1**. *Minimum Evolution under Least Square (LS)*

min⁡(X,v)‖v‖1s.t.f(D,X,v)=0X∈X
 MathType@MTEF@5@5@+=feaafiart1ev1aaatCvAUfKttLearuWrP9MDH5MBPbIqV92AaeXatLxBI9gBaebbnrfifHhDYfgasaacPC6xNi=xI8qiVKYPFjYdHaVhbbf9v8qqaqFr0xc9vqFj0dXdbba91qpepeI8k8fiI+fsY=rqGqVepae9pg0db9vqaiVgFr0xfr=xfr=xc9adbaqaaeGacaGaaiaabeqaaeqabiWaaaGcbaqbaeqabmGaaaqaamaaxababaGagiyBa0MaeiyAaKMaeiOBa4galeaacqGGOaakieqacqWFybawcqGGSaalcqWF2bGDcqGGPaqkaeqaaaGcbaWaauWaaeaacqWF2bGDaiaawMa7caGLkWoadaWgaaWcbaGaeGymaedabeaaaOqaaiabdohaZjabc6caUiabdsha0jabc6caUaqaaiabdAgaMjabcIcaOiab=reaejabcYcaSiab=HfayjabcYcaSiab=zha2jabcMcaPiabg2da9iabicdaWaqaaaqaaiab=HfayjabgIGioprtHrhAL1wy0L2yHvtyaeHbnfgDOvwBHrxAJfwnaGabaiab+Dr8ybaaaaa@5909@

where || · ||_1 _is the ℒ1
 MathType@MTEF@5@5@+=feaafiart1ev1aaatCvAUfKttLearuWrP9MDH5MBPbIqV92AaeXatLxBI9gBaebbnrfifHhDYfgasaacPC6xNi=xH8viVGI8Gi=hEeeu0xXdbba9frFj0xb9qqpG0dXdb9aspeI8k8fiI+fsY=rqGqVepae9pg0db9vqaiVgFr0xfr=xfr=xc9adbaqaaeGacaGaaiaabeqaaeqabiWaaaGcbaWenfgDOvwBHrxAJfwnHbqeg0uy0HwzTfgDPnwy1aaceaGae8NeHW0aaWbaaSqabeaacqaIXaqmaaaaaa@37B1@-vector norm; **v **is a vector of the 2*n *- 3 edge lengths; **X **is a *n*(*n *- 1)/2 × (2*n *- 3) *topological matrix*[4] encoding a phylogenetic tree as an unrooted binary tree with the *n *taxa in Γ as terminal vertices (*leaves*); X
 MathType@MTEF@5@5@+=feaafiart1ev1aaatCvAUfKttLearuWrP9MDH5MBPbIqV92AaeXatLxBI9gBaebbnrfifHhDYfgasaacPC6xNi=xH8viVGI8Gi=hEeeu0xXdbba9frFj0xb9qqpG0dXdb9aspeI8k8fiI+fsY=rqGqVepae9pg0db9vqaiVgFr0xfr=xfr=xc9adbaqaaeGacaGaaiaabeqaaeqabiWaaaGcbaWenfgDOvwBHrxAJfwnHbqeg0uy0HwzTfgDPnwy1aaceaGae83fXJfaaa@3775@ is the set of all the topological matrices; finally, *f*(· , · , ·) defines the level of compatibility among the distances in **D **and the distances induced by the phylogenetic tree edges. Any *optimal solution *(X*, v*) of problem (1) defines a phylogenetic tree satisfying the minimum evolution principle. A topological matrix **X **is an *Edge-Path incidence matrix of a Tree *(EPT) (see [5], and additional files 1 and 2) that encodes a tree as follows: any generic entry *x*_*ij,k *_is set to 1 if the edge *k *belongs to the path from the leaf *i *to the leaf *j*, 0 otherwise. In the rest of the paper we refer to problem (1) as the *ME problem*.

The distance matrix **D **of problem (1) is estimated from the dataset, e.g., accordingly to any method described in [6-12]. Condition *f*(**D**, **X**, **v**) = 0 typically imposes that, for any given EPT matrix **X**, **v **minimizes the (weighted) sum of the square values of the differences between the distances in **D **and the corresponding distances induced by the phylogenetic tree edges [6,13]. In particular, under the unweighted least-square (also called Ordinary Least-Squares (OLS)) [2]:

**v **= **X**^†^**D**^Δ^

where **X**^† ^is the Moore-Penrose pseudoinverse of **X**, and **D**^Δ ^is a vector whose components are obtained by taking row per row the entries of the strictly upper triangular matrix of **D**.

Others [14] and [15] have suggested the use of a Weighted Least-Squares (WLS) function:

**v **= (**X**^*t*^**WX**)^-1^**X**^*t*^**WD**^Δ^

where **W **is a strictly positive definite diagonal matrix whose entries *w*_*ij *_represent weights associated to leaves *i *and *j*. Finally, Hasegawa *et al*. [16] introduced a Generalized Least-Squares (GLS) function in which **v **is computed using:

**v **= (**X**^*t*^**C**^-1^**X**)^-1^**X**^*t*^**C**^-1^**D**^Δ^

where **C **is a strictly positive definite symmetric matrix representing the covariance matrix of **D**. To avoid the occurrence of negative branch lengths [14,17], problem (1) can be modified as follows:

**Problem 2**. *Minimum Evolution under Linear Programming (LP)*

min⁡(X,v)‖v‖1s.t.Xv≥DΔv∈ℝ+2n−3X∈X
 MathType@MTEF@5@5@+=feaafiart1ev1aaatCvAUfKttLearuWrP9MDH5MBPbIqV92AaeXatLxBI9gBaebbnrfifHhDYfgasaacPC6xNi=xI8qiVKYPFjYdHaVhbbf9v8qqaqFr0xc9vqFj0dXdbba91qpepeI8k8fiI+fsY=rqGqVepae9pg0db9vqaiVgFr0xfr=xfr=xc9adbaqaaeGacaGaaiaabeqaaeqabiWaaaGcbaqbaeaabqabaaaaaeaacyGGTbqBcqGGPbqAcqGGUbGBdaWgaaWcbaGaeiikaGccbeGae8hwaGLaeiilaWIae8NDayNaeiykaKcabeaaaOqaamaafmaabaGae8NDayhacaGLjWUaayPcSdWaaSbaaSqaaiabigdaXaqabaaakeaaaeaaaeaaieGacqGFZbWCcqGFUaGlcqGF0baDcqGFUaGlaeaacqWFybawcqWF2bGDaeaacqGHLjYSaeaacqWFebardaahaaWcbeqaaiabfs5aebaaaOqaaaqaaiab=zha2bqaaiabgIGiodqaamrr1ngBPrwtHrhAYaqeguuDJXwAKbstHrhAGq1DVbaceaGae0xhHi1aa0baaSqaaiabgUcaRaqaaiabikdaYiabd6gaUjabgkHiTiabiodaZaaaaOqaaaqaaiab=HfaybqaaiabgIGiodqaamrtHrhAL1wy0L2yHvtyaeXbnfgDOvwBHrxAJfwnaGqbaiab8Dr8ybaaaaa@68A4@

Unfortunately, both problems (1) and (2) are NP
 MathType@MTEF@5@5@+=feaafiart1ev1aaatCvAUfKttLearuWrP9MDH5MBPbIqV92AaeXatLxBI9gBaebbnrfifHhDYfgasaacPC6xNi=xH8viVGI8Gi=hEeeu0xXdbba9frFj0xb9qqpG0dXdb9aspeI8k8fiI+fsY=rqGqVepae9pg0db9vqaiVgFr0xfr=xfr=xc9adbaqaaeGacaGaaiaabeqaaeqabiWaaaGcbaWenfgDOvwBHrxAJfwnHbqeg0uy0HwzTfgDPnwy1aaceaGae8xdX7Kaeeiuaafaaa@3888@-hard [18]. In this context, let us observe that, given Γ, the cardinality of X
 MathType@MTEF@5@5@+=feaafiart1ev1aaatCvAUfKttLearuWrP9MDH5MBPbIqV92AaeXatLxBI9gBaebbnrfifHhDYfgasaacPC6xNi=xH8viVGI8Gi=hEeeu0xXdbba9frFj0xb9qqpG0dXdb9aspeI8k8fiI+fsY=rqGqVepae9pg0db9vqaiVgFr0xfr=xfr=xc9adbaqaaeGacaGaaiaabeqaaeqabiWaaaGcbaWenfgDOvwBHrxAJfwnHbqeg0uy0HwzTfgDPnwy1aaceaGae83fXJfaaa@3775@ is:

|X
 MathType@MTEF@5@5@+=feaafiart1ev1aaatCvAUfKttLearuWrP9MDH5MBPbIqV92AaeXatLxBI9gBaebbnrfifHhDYfgasaacPC6xNi=xH8viVGI8Gi=hEeeu0xXdbba9frFj0xb9qqpG0dXdb9aspeI8k8fiI+fsY=rqGqVepae9pg0db9vqaiVgFr0xfr=xfr=xc9adbaqaaeGacaGaaiaabeqaaeqabiWaaaGcbaWenfgDOvwBHrxAJfwnHbqeg0uy0HwzTfgDPnwy1aaceaGae83fXJfaaa@3775@| = (2|Γ| - 5)!! = (2*n *- 5)!!

where *n*!! is the double factorial of *n*. Hence, the number of topological matrices grows exponentially with the number of leaves ([6], p. 25, and see additional files 1 and 2).

Problem (1) has received great attention from the scientific community such that exact and approximate algorithms to solve it have been developed. Exact algorithms for solving problem (1) are typically based on an exhaustive approach (i.e., enumerating all possible trees **X**). As an example, PAUP* 4.0 [19] allows exhaustive search for datasets containing up to 12 taxa. A number of heuristics were also developed in the last 20 years. E.g., Rzhetsky and Nei [2,3] (i) start from a Neighbor-Joining (NJ) tree [20,21], (ii) apply a local search generating topologies within a given *topological distance *(see [2]) from the NJ tree, and (iii) return the best topology found. Kumar [22] further improved the approach as follows: starting from a topology, a leaf *l *is selects at each step and all possible assignments of *l *on the topology are tested. Despite that the neighborhood size in Kumar's approach is larger than in Rzhetsky and Nei's algorithm, it requires examining a number of topologies that is, at most, an exponential function of the number of leaves *n*: (*n *- 1)!/2, and generates solution in a shorter computing time. Finally, Bryant and Waddell [4] implemented programming optimisation and Desper and Gascuel [23] introduced a greedy search that both improved speed and accuracy of the search.

Here, we introduce the Ant Colony Optimization (ACO) algorithm for estimating phylogenies under the minimum evolution principle, and show that ACO has the potential to compete with other widely-used methods. ACO (see [24,25] for an introduction, and [26,27] for recent reviews) is a widely-used metaheuristic approach for solving hard combinatorial optimization problems. ACO is inspired from the pheromone trail laying and following behavior of real ants. ACO implements indirect communication among simple agents, called (artificial) ants. Communication is mediated by (artificial) pheromone trails implemented as a probabilistic model to which the ants adapt during the algorithm's execution to reflect their search experience. ACO has proven a successful technique for numerous NP
 MathType@MTEF@5@5@+=feaafiart1ev1aaatCvAUfKttLearuWrP9MDH5MBPbIqV92AaeXatLxBI9gBaebbnrfifHhDYfgasaacPC6xNi=xH8viVGI8Gi=hEeeu0xXdbba9frFj0xb9qqpG0dXdb9aspeI8k8fiI+fsY=rqGqVepae9pg0db9vqaiVgFr0xfr=xfr=xc9adbaqaaeGacaGaaiaabeqaaeqabiWaaaGcbaWenfgDOvwBHrxAJfwnHbqeg0uy0HwzTfgDPnwy1aaceaGae8xdX7Kaeeiuaafaaa@3888@-hard combinatorial optimization problems (see [28]), although no application to the ME phylogeny problem is currently known. Our specific implementation of the ACO algorithm exploits a stochastic version of the Neighbor-Joining (NJ) algorithm [20,21] to explore tree space.

## Results and Discussion

### Iterative addition

Given a set Γ of taxa, let us define a *partial tree *as a *m*-leaf tree whose leaves are taxa of a subset Γ*' *⊂ Γ, with *m *= |Γ*'*|. Moreover, given a partial tree, with node set *V *and edge set *E*, let us say that we *add/insert a leaf i *(not yet in Γ*'*) on the edge (*r*, *s*) ∈ *E *(i.e., the edge joining the nodes *r*, *s *∈ *V*), and generate a new partial tree with node set V^
 MathType@MTEF@5@5@+=feaafiart1ev1aaatCvAUfKttLearuWrP9MDH5MBPbIqV92AaeXatLxBI9gBaebbnrfifHhDYfgasaacPC6xNi=xH8viVGI8Gi=hEeeu0xXdbba9frFj0xb9qqpG0dXdb9aspeI8k8fiI+fsY=rqGqVepae9pg0db9vqaiVgFr0xfr=xfr=xc9adbaqaaeGacaGaaiaabeqaaeqabiWaaaGcbaGafmOvayLbaKaaaaa@2D18@ = *V *∪ {*i*, *t*} and edge set E^
 MathType@MTEF@5@5@+=feaafiart1ev1aaatCvAUfKttLearuWrP9MDH5MBPbIqV92AaeXatLxBI9gBaebbnrfifHhDYfgasaacPC6xNi=xH8viVGI8Gi=hEeeu0xXdbba9frFj0xb9qqpG0dXdb9aspeI8k8fiI+fsY=rqGqVepae9pg0db9vqaiVgFr0xfr=xfr=xc9adbaqaaeGacaGaaiaabeqaaeqabiWaaaGcbaGafmyrauKbaKaaaaa@2CF6@ = *E *∪ {(*r*, *t*), (*t*, *s*), (*t*, *i*)}\{(*r*, *s*)}. In other words, we add a leaf *i *on an edge, divide that edge with a new node *t*, and join the leaf *i *to *t*. All algorithms described here build complete phylogenetic trees by iteratively adding one leaf at a time on the edges of a partial tree.

### Primal bound

To generate a first upper bound [5] of the ME problem, we adapted the *Sequential Addition *(SA) greedy algorithm [6]. The Sequential Addition algorithm is less prone, than NJ, to generate a systematic local optimum at the end of the search (i.e., starting from "too good" a primal bound may lead to inefficient results [29]).

**Figure 1:**
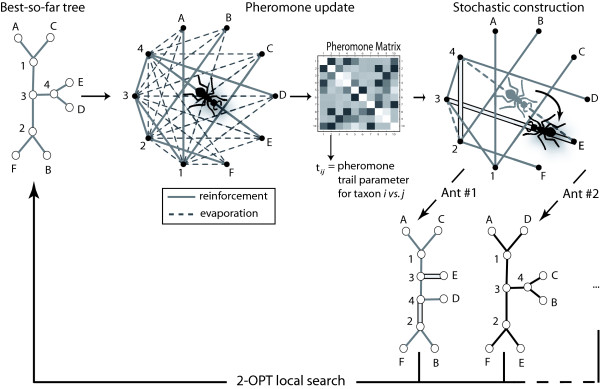
Principle of the ACO-ME algorithm. The core iteration includes three main steps: (i) the pheromone update phase during which artificial ants walk on a graph with all possible connections among the *n *taxa and (*n *- 2) internal nodes, and lay a trail of volatile pheromone on the branches of the starting tree; (ii) the stochastic construction phase during which new trees are built using both the heuristic information of the pairwise distances and the stochastic process guided by the newly-updated pheromone trail matrix (ants follow a given edge with a probability which is a function of the amount of pheromone on that edge); and (iii) the 2-OPT local search phase that corresponds to a local search using taxon swapping. The curved arrow indicates the stochastic jump of an ant from one edge to another. See text for details.

The pseudo-code of our version of the Sequential Addition algorithm is presented in Figure 1. In the initialization step, we arbitrarily chose a subset Γ*' *⊆ Γ of *m *≤ *n *leaves, and we generate as initial *m*-leaf partial tree, i.e., an optimal solution of the problem (1) when only *m *leaves are considered. A each iteration, we join the leaf *i *to all possible leaves already present in Γ*'*, and choose the solution that minimize tree length (we break possible ties randomly), hence, generating a new partial tree and new set Γ*' *= Γ*' *∪ {*i*}. We iterate the procedure until a tree with *n *leaves is obtained. Finally, fixing the topology matrix X^m
 MathType@MTEF@5@5@+=feaafiart1ev1aaatCvAUfKttLearuWrP9MDH5MBPbIqV92AaeXatLxBI9gBaebbnrfifHhDYfgasaacPC6xNi=xH8viVGI8Gi=hEeeu0xXdbba9frFj0xb9qqpG0dXdb9aspeI8k8fiI+fsY=rqGqVepae9pg0db9vqaiVgFr0xfr=xfr=xc9adbaqaaeGacaGaaiaabeqaaeqabiWaaaGcbaacbeGaf8hwaGLbaKaadaWgaaWcbaGaemyBa0gabeaaaaa@2EB1@, we determine the optimal edge weights by imposing *f *(**D**, X^m
 MathType@MTEF@5@5@+=feaafiart1ev1aaatCvAUfKttLearuWrP9MDH5MBPbIqV92AaeXatLxBI9gBaebbnrfifHhDYfgasaacPC6xNi=xH8viVGI8Gi=hEeeu0xXdbba9frFj0xb9qqpG0dXdb9aspeI8k8fiI+fsY=rqGqVepae9pg0db9vqaiVgFr0xfr=xfr=xc9adbaqaaeGacaGaaiaabeqaaeqabiWaaaGcbaacbeGaf8hwaGLbaKaadaWgaaWcbaGaemyBa0gabeaaaaa@2EB1@, **v **= 0, and return the length of the tree, i.e., the upper bound on the optimal solution of the ME problem.

Unfortunately, the computation complexity of our heuristic is *O*((2*m *- 5)!! + *n*(*n - m*)^2^). AT each iteration, given a partial tree, i.e., a *k*-leaf phylogenetic tree of the leaves in Γ*' *⊂ Γ with *k *= |Γ*'*|, and a leaf *i *not in Γ*'*, the procedure generates all the different (*k *+ 1)-leaf partial trees that can be obtained by adding the leaf *i *in each edge of the current partial tree.

### The ant colony optimization algorithm

**Figure 2:**
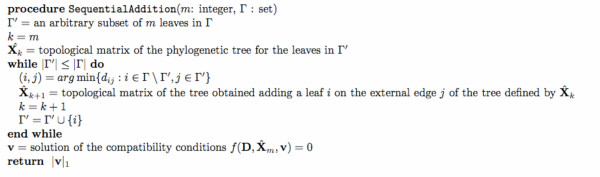
High-level pseudo-code for the Sequential Addition heuristic.

The specific ACO algorithm for the minimum evolution problem (hereafter ACO-ME) that we introduce here (cf. pseudo-code in Figure 2), is a hybrid between the Max-Min Ant System (MMAS) [30,31] and the Approximate Nondeterministic Tree Search (ANTS) [32]. Both methods are modifications of the original Ant System approach [33].

The core of the ACO-ME algorithm is the iteration phase, where the ants generate a set T
 MathType@MTEF@5@5@+=feaafiart1ev1aaatCvAUfKttLearuWrP9MDH5MBPbIqV92AaeXatLxBI9gBaebbnrfifHhDYfgasaacPC6xNi=xH8viVGI8Gi=hEeeu0xXdbba9frFj0xb9qqpG0dXdb9aspeI8k8fiI+fsY=rqGqVepae9pg0db9vqaiVgFr0xfr=xfr=xc9adbaqaaeGacaGaaiaabeqaaeqabiWaaaGcbaWenfgDOvwBHrxAJfwnHbqeg0uy0HwzTfgDPnwy1aaceaGae83eXtfaaa@376D@ of trees. Then, starting from the trees in T
 MathType@MTEF@5@5@+=feaafiart1ev1aaatCvAUfKttLearuWrP9MDH5MBPbIqV92AaeXatLxBI9gBaebbnrfifHhDYfgasaacPC6xNi=xH8viVGI8Gi=hEeeu0xXdbba9frFj0xb9qqpG0dXdb9aspeI8k8fiI+fsY=rqGqVepae9pg0db9vqaiVgFr0xfr=xfr=xc9adbaqaaeGacaGaaiaabeqaaeqabiWaaaGcbaWenfgDOvwBHrxAJfwnHbqeg0uy0HwzTfgDPnwy1aaceaGae83eXtfaaa@376D@, a local search is performed until a locally optimal tree is found and compared with the current-best tree. If stopping conditions are met the procedure ends, otherwise the iteration phase is repeated.

Each ant builds a phylogenetic tree by iteratively adding a leaf at a time to a partial tree. Following a *relation-learning *model [34], the choices performed by an ant about (i) which leaf to insert, and (ii) where to add it on the partial tree are based of a set of parameters {*τ*_*ij*_} called *pheromone trails*. The values of the pheromone trail parameters {*τ*_*ij*_} represent a stochastic desirability that a leaf *i *shares a direct common ancestor with a vertex *j *on a partial tree. The ants generate a new set T
 MathType@MTEF@5@5@+=feaafiart1ev1aaatCvAUfKttLearuWrP9MDH5MBPbIqV92AaeXatLxBI9gBaebbnrfifHhDYfgasaacPC6xNi=xH8viVGI8Gi=hEeeu0xXdbba9frFj0xb9qqpG0dXdb9aspeI8k8fiI+fsY=rqGqVepae9pg0db9vqaiVgFr0xfr=xfr=xc9adbaqaaeGacaGaaiaabeqaaeqabiWaaaGcbaWenfgDOvwBHrxAJfwnHbqeg0uy0HwzTfgDPnwy1aaceaGae83eXtfaaa@376D@ of trees and the pheromone trail parameters are updated at the end of the main iteration phase.

Let us now consider the algorithm in more details. It uses two identical data structures: s* and *s*_*k*_. The former stores the current-best complete *reconstruction *(solution) known, whereas the latter stores the best complete reconstruction obtained by the ants during iteration *k*. The algorithm also uses a variable *n*_*a*_, i.e., the number of artificial ants. How to set the value of *n*_*a *_is discussed in the Parameter settings section. In the initialization phase, s* is first set to the reconstruction obtained by the Sequential Addition algorithm and *s*_*k *_is set to null, then the pheromone trail parameters are updated. We implemented the MMAS [30,31] method of pheromone update, where *τ*_*min *_≤ *τ*_*ij *_≤ *τ*_*max*_. Here, we set *τ*_*min *_and *τ*_*max *_to 0.0001 and 0.9999, respectively [35]. In the initialization phase, the pheromone trail parameters {*τ*_*ij*_} are set to 0.5, i.e., all positions for leaf insertion have the same desirability.

Before describing the iteration phase, let us introduce some definitions. Let Gk
 MathType@MTEF@5@5@+=feaafiart1ev1aaatCvAUfKttLearuWrP9MDH5MBPbIqV92AaeXatLxBI9gBaebbnrfifHhDYfgasaacPC6xNi=xH8viVGI8Gi=hEeeu0xXdbba9frFj0xb9qqpG0dXdb9aspeI8k8fiI+fsY=rqGqVepae9pg0db9vqaiVgFr0xfr=xfr=xc9adbaqaaeGacaGaaiaabeqaaeqabiWaaaGcbaWenfgDOvwBHrxAJfwnHbqeg0uy0HwzTfgDPnwy1aaceaGae8NbXF0aaSbaaSqaaiabdUgaRbqabaaaaa@38DE@ be a partial tree with *k *leaves, *V*(Gk
 MathType@MTEF@5@5@+=feaafiart1ev1aaatCvAUfKttLearuWrP9MDH5MBPbIqV92AaeXatLxBI9gBaebbnrfifHhDYfgasaacPC6xNi=xH8viVGI8Gi=hEeeu0xXdbba9frFj0xb9qqpG0dXdb9aspeI8k8fiI+fsY=rqGqVepae9pg0db9vqaiVgFr0xfr=xfr=xc9adbaqaaeGacaGaaiaabeqaaeqabiWaaaGcbaWenfgDOvwBHrxAJfwnHbqeg0uy0HwzTfgDPnwy1aaceaGae8NbXF0aaSbaaSqaaiabdUgaRbqabaaaaa@38DE@) the set of vertices of Gk
 MathType@MTEF@5@5@+=feaafiart1ev1aaatCvAUfKttLearuWrP9MDH5MBPbIqV92AaeXatLxBI9gBaebbnrfifHhDYfgasaacPC6xNi=xH8viVGI8Gi=hEeeu0xXdbba9frFj0xb9qqpG0dXdb9aspeI8k8fiI+fsY=rqGqVepae9pg0db9vqaiVgFr0xfr=xfr=xc9adbaqaaeGacaGaaiaabeqaaeqabiWaaaGcbaWenfgDOvwBHrxAJfwnHbqeg0uy0HwzTfgDPnwy1aaceaGae8NbXF0aaSbaaSqaaiabdUgaRbqabaaaaa@38DE@, and ΓGk
 MathType@MTEF@5@5@+=feaafiart1ev1aaatCvAUfKttLearuWrP9MDH5MBPbIqV92AaeXatLxBI9gBaebbnrfifHhDYfgasaacPC6xNi=xH8viVGI8Gi=hEeeu0xXdbba9frFj0xb9qqpG0dXdb9aspeI8k8fiI+fsY=rqGqVepae9pg0db9vqaiVgFr0xfr=xfr=xc9adbaqaaeGacaGaaiaabeqaaeqabiWaaaGcbaGaeu4KdC0aaSbaaSqaamrtHrhAL1wy0L2yHvtyaeHbnfgDOvwBHrxAJfwnaGabaiab=zq8hnaaBaaameaacqWGRbWAaeqaaaWcbeaaaaa@3A7E@ the set of leaves of Gk
 MathType@MTEF@5@5@+=feaafiart1ev1aaatCvAUfKttLearuWrP9MDH5MBPbIqV92AaeXatLxBI9gBaebbnrfifHhDYfgasaacPC6xNi=xH8viVGI8Gi=hEeeu0xXdbba9frFj0xb9qqpG0dXdb9aspeI8k8fiI+fsY=rqGqVepae9pg0db9vqaiVgFr0xfr=xfr=xc9adbaqaaeGacaGaaiaabeqaaeqabiWaaaGcbaWenfgDOvwBHrxAJfwnHbqeg0uy0HwzTfgDPnwy1aaceaGae8NbXF0aaSbaaSqaaiabdUgaRbqabaaaaa@38DE@. Let us also use the *recursive distance *definition of [23,36]: if A and B are two non-intersecting subtrees from a tree G
 MathType@MTEF@5@5@+=feaafiart1ev1aaatCvAUfKttLearuWrP9MDH5MBPbIqV92AaeXatLxBI9gBaebbnrfifHhDYfgasaacPC6xNi=xH8viVGI8Gi=hEeeu0xXdbba9frFj0xb9qqpG0dXdb9aspeI8k8fiI+fsY=rqGqVepae9pg0db9vqaiVgFr0xfr=xfr=xc9adbaqaaeGacaGaaiaabeqaaeqabiWaaaGcbaWenfgDOvwBHrxAJfwnHbqeg0uy0HwzTfgDPnwy1aaceaGae8NbXFeaaa@3753@, then the average distance between A and B is:

ΔA|B=1|A||B|∑i∈A,j∈Bdij.
 MathType@MTEF@5@5@+=feaafiart1ev1aaatCvAUfKttLearuWrP9MDH5MBPbIqV92AaeXatLxBI9gBaebbnrfifHhDYfgasaacPC6xNi=xI8qiVKYPFjYdHaVhbbf9v8qqaqFr0xc9vqFj0dXdbba91qpepeI8k8fiI+fsY=rqGqVepae9pg0db9vqaiVgFr0xfr=xfr=xc9adbaqaaeGacaGaaiaabeqaaeqabiWaaaGcbaGaeuiLdq0aaSbaaSqaaiabdgeabjabcYha8jabdkeacbqabaGccqGH9aqpjuaGdaWcaaqaaiabigdaXaqaaiabcYha8jabdgeabjabcYha8jabcYha8jabdkeacjabcYha8baadaaeqbqaaiabdsgaKnaaBaaabaGaemyAaKMaemOAaOgabeaaaeaacqWGPbqAcqGHiiIZcqWGbbqqcqGGSaalcqWGQbGAcqGHiiIZcqWGcbGqaeqacqGHris5aiabc6caUaaa@4BD2@

**Figure 3:**
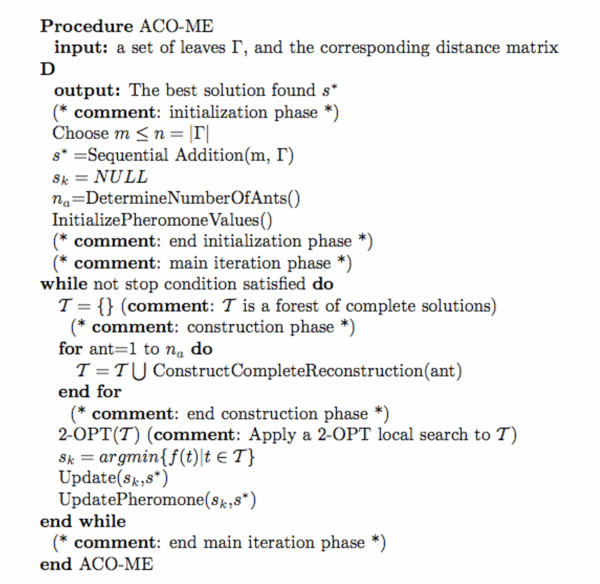
High-level pseudo-code for the ACO algorithm.

In the iteration phase, each artificial ant *r *generates a complete phylogenetic tree using the ConstructCompleteReconstruction(*r*) procedure, as illustrated in Figure 3: ant *r *randomly selects four leaves from the set Γ, and builds a partial tree Gk
 MathType@MTEF@5@5@+=feaafiart1ev1aaatCvAUfKttLearuWrP9MDH5MBPbIqV92AaeXatLxBI9gBaebbnrfifHhDYfgasaacPC6xNi=xH8viVGI8Gi=hEeeu0xXdbba9frFj0xb9qqpG0dXdb9aspeI8k8fiI+fsY=rqGqVepae9pg0db9vqaiVgFr0xfr=xfr=xc9adbaqaaeGacaGaaiaabeqaaeqabiWaaaGcbaWenfgDOvwBHrxAJfwnHbqeg0uy0HwzTfgDPnwy1aaceaGae8NbXF0aaSbaaSqaaiabdUgaRbqabaaaaa@38DE@, *k *= 4, then, ant *r *(i) chooses, among the leaves not yet inserted in the partial topology, the leaf *i *defining the smallest distance *d*_*ij*_, *j *∈ ΓGk
 MathType@MTEF@5@5@+=feaafiart1ev1aaatCvAUfKttLearuWrP9MDH5MBPbIqV92AaeXatLxBI9gBaebbnrfifHhDYfgasaacPC6xNi=xH8viVGI8Gi=hEeeu0xXdbba9frFj0xb9qqpG0dXdb9aspeI8k8fiI+fsY=rqGqVepae9pg0db9vqaiVgFr0xfr=xfr=xc9adbaqaaeGacaGaaiaabeqaaeqabiWaaaGcbaGaeu4KdC0aaSbaaSqaamrtHrhAL1wy0L2yHvtyaeHbnfgDOvwBHrxAJfwnaGabaiab=zq8hnaaBaaameaacqWGRbWAaeqaaaWcbeaaaaa@3A7E@, and (ii) computes the probability that *i *has a common ancestor with the vertex *j *∈ *V *(Tk
 MathType@MTEF@5@5@+=feaafiart1ev1aaatCvAUfKttLearuWrP9MDH5MBPbIqV92AaeXatLxBI9gBaebbnrfifHhDYfgasaacPC6xNi=xH8viVGI8Gi=hEeeu0xXdbba9frFj0xb9qqpG0dXdb9aspeI8k8fiI+fsY=rqGqVepae9pg0db9vqaiVgFr0xfr=xfr=xc9adbaqaaeGacaGaaiaabeqaaeqabiWaaaGcbaWenfgDOvwBHrxAJfwnHbqeg0uy0HwzTfgDPnwy1aaceaGae83eXt1aaSbaaSqaaiabdUgaRbqabaaaaa@38F8@) using the formula suggested by ANTS [32]:

pij=ατij+(1−α)ηij∑q∈Γ\ΓGk[ατqj+(1−α)ηqj]
 MathType@MTEF@5@5@+=feaafiart1ev1aaatCvAUfKttLearuWrP9MDH5MBPbIqV92AaeXatLxBI9gBaebbnrfifHhDYfgasaacPC6xNi=xI8qiVKYPFjYdHaVhbbf9v8qqaqFr0xc9vqFj0dXdbba91qpepeI8k8fiI+fsY=rqGqVepae9pg0db9vqaiVgFr0xfr=xfr=xc9adbaqaaeGacaGaaiaabeqaaeqabiWaaaGcbaGaemiCaa3aaSbaaSqaaiabdMgaPjabdQgaQbqabaGccqGH9aqpjuaGdaWcaaqaaGGaciab=f7aHjab=r8a0naaBaaabaGaemyAaKMaemOAaOgabeaacqGHRaWkcqGGOaakcqaIXaqmcqGHsislcqWFXoqycqGGPaqkcqWF3oaAdaWgaaqaaiabdMgaPjabdQgaQbqabaaabaWaaabeaeaacqGGBbWwcqWFXoqycqWFepaDdaWgaaqaaiabdghaXjabdQgaQbqabaGaey4kaSIaeiikaGIaeGymaeJaeyOeI0Iae8xSdeMaeiykaKIae83TdG2aaSbaaeaacqWGXbqCcqWGQbGAaeqaaiabc2faDbqaaiabdghaXjabgIGiolabfo5ahjabcYfaCjabfo5ahnaaBaaabaWenfgDOvwBHrxAJfwnHbqeg0uy0HwzTfgDPnwy1aaceaGae4NbXF0aaSbaaeaacqWGRbWAaeqaaaqabaaabeGaeyyeIuoaaaaaaa@6C2E@

where *η*_*ij *_represents the "heuristic desirability" that leaf *i *shares a common ancestor with a vertex *j *of *V*(Gk
 MathType@MTEF@5@5@+=feaafiart1ev1aaatCvAUfKttLearuWrP9MDH5MBPbIqV92AaeXatLxBI9gBaebbnrfifHhDYfgasaacPC6xNi=xH8viVGI8Gi=hEeeu0xXdbba9frFj0xb9qqpG0dXdb9aspeI8k8fiI+fsY=rqGqVepae9pg0db9vqaiVgFr0xfr=xfr=xc9adbaqaaeGacaGaaiaabeqaaeqabiWaaaGcbaWenfgDOvwBHrxAJfwnHbqeg0uy0HwzTfgDPnwy1aaceaGae8NbXF0aaSbaaSqaaiabdUgaRbqabaaaaa@38DE@) (whereas *τ*_*ij *_represents the corresponding "stochastic desirability"). Finally, *α *∈ [01] allows the relative weighting of heuristic and stochastic desirabilities. The heuristic desirability *η*_*ij *_is computed as:

*η*_*ij *_= (Δ_*ij *_- *u*_*i *_- *u*_*j*_)^-1^

where ui=∑j∉VGkΔij/(|VΓGk|)
 MathType@MTEF@5@5@+=feaafiart1ev1aaatCvAUfKttLearuWrP9MDH5MBPbIqV92AaeXatLxBI9gBaebbnrfifHhDYfgasaacPC6xNi=xH8viVGI8Gi=hEeeu0xXdbba9frFj0xb9qqpG0dXdb9aspeI8k8fiI+fsY=rqGqVepae9pg0db9vqaiVgFr0xfr=xfr=xc9adbaqaaeGacaGaaiaabeqaaeqabiWaaaGcbaGaemyDau3aaSbaaSqaaiabdMgaPbqabaGccqGH9aqpdaaeqaqaaiabfs5aenaaBaaaleaacqWGPbqAcqWGQbGAaeqaaOGaei4la8IaeiikaGIaeiiFaWNaemOvay1aaSbaaSqaaiabfo5ahnaaBaaameaat0uy0HwzTfgDPnwy1egaryqtHrhAL1wy0L2yHvdaiqaacqWFge=rdaWgaaqaaiabdUgaRbqabaaabeaaaSqabaGccqGG8baFcqGGPaqkaSqaaiabdQgaQjabgMGiplabdAfawnaaBaaameaacqWFge=rdaWgaaqaaiabdUgaRbqabaaabeaaaSqab0GaeyyeIuoaaaa@534A@, i.e., the sum of the distances from i to the leaves not yet inserted in the partial tree divided the number of leaves inserted in the partial tree.

Note that *η*_*ij*_, Δ_*ij*_, and *u*_*i *_correspond to the quantities used in the Neighbor-Joining algorithm[20,21](see also[23]). Hence, computation of the vector **p**_*i *_= {*p*_*ij*_}, for all *i *∈ Γ, can be interpreted as the stochastic application of the Neighbor-Joining algorithm. A possible problem (not observed yet in practice in our analyses) is that *η*_*ij *_can take negative values. Finally, ant *r *randomly chooses a vertex *j *on the basis of the probabilities **p**_*i*_, and the leaf *i *is added to the tree.

At the end of the construction phase, a set T
 MathType@MTEF@5@5@+=feaafiart1ev1aaatCvAUfKttLearuWrP9MDH5MBPbIqV92AaeXatLxBI9gBaebbnrfifHhDYfgasaacPC6xNi=xH8viVGI8Gi=hEeeu0xXdbba9frFj0xb9qqpG0dXdb9aspeI8k8fiI+fsY=rqGqVepae9pg0db9vqaiVgFr0xfr=xfr=xc9adbaqaaeGacaGaaiaabeqaaeqabiWaaaGcbaWenfgDOvwBHrxAJfwnHbqeg0uy0HwzTfgDPnwy1aaceaGae83eXtfaaa@376D@ of trees is obtained and a 2-OPT local search (with best-improvement and without candidate list [37,38]) is iteratively performed on each tree: two randomly-chosen leaves are swapped and the tree length is evaluated. Swap i is performed on the new tree if swap i-1 generated an improvement, otherwise it is performed on the old tree. To reduce the 2-OPT computational overhead, we perform no more than 10 swappings on each tree in T
 MathType@MTEF@5@5@+=feaafiart1ev1aaatCvAUfKttLearuWrP9MDH5MBPbIqV92AaeXatLxBI9gBaebbnrfifHhDYfgasaacPC6xNi=xH8viVGI8Gi=hEeeu0xXdbba9frFj0xb9qqpG0dXdb9aspeI8k8fiI+fsY=rqGqVepae9pg0db9vqaiVgFr0xfr=xfr=xc9adbaqaaeGacaGaaiaabeqaaeqabiWaaaGcbaWenfgDOvwBHrxAJfwnHbqeg0uy0HwzTfgDPnwy1aaceaGae83eXtfaaa@376D@. If the best tree generated by the 2-OPT local search is shorter than the tree in s*, both s* and *s*_*k *_are updated, otherwise only *s*_*k *_is updated.

The pheromone update completes the iteration phase: each entry *τ*_*ij *_is updated following:

*τ*_*ij *_← (1 - *ρ*)*τ*_*ij *_+ *ε*_*ij*_

where

εij={κρ/lG|Γ|,if wi adjacent to wj in sbest;0,otherwise.
 MathType@MTEF@5@5@+=feaafiart1ev1aaatCvAUfKttLearuWrP9MDH5MBPbIqV92AaeXatLxBI9gBaebbnrfifHhDYfgasaacPC6xNi=xI8qiVKYPFjYdHaVhbbf9v8qqaqFr0xc9vqFj0dXdbba91qpepeI8k8fiI+fsY=rqGqVepae9pg0db9vqaiVgFr0xfr=xfr=xc9adbaqaaeGacaGaaiaabeqaaeqabiWaaaGcbaacciGae8xTdu2aaSbaaSqaaiabdMgaPjabdQgaQbqabaGccqGH9aqpdaGabeqaauaabaqaciaaaeaacqWF6oWAcqWFbpGCcqGGVaWlcqWGSbaBdaWgaaWcbaWenfgDOvwBHrxAJfwnHbqeg0uy0HwzTfgDPnwy1aaceaGae4NbXF0aaSbaaWqaamaaemaabaGaeu4KdCeacaGLhWUaayjcSdaabeaaaSqabaGccqGGSaalaeaacqqGPbqAcqqGMbGzcqqGGaaicqWG3bWDdaWgaaWcbaGaemyAaKgabeaakiabbccaGiabbggaHjabbsgaKjabbQgaQjabbggaHjabbogaJjabbwgaLjabb6gaUjabbsha0jabbccaGiabbsha0jabb+gaVjabbccaGiabdEha3naaBaaaleaacqWGQbGAaeqaaOGaeeiiaaIaeeyAaKMaeeOBa4MaeeiiaaIaem4Cam3aaWbaaSqabeaacqWGIbGycqWGLbqzcqWGZbWCcqWG0baDaaGccqGG7aWoaeaacqaIWaamcqGGSaalaeaacqqGVbWBcqqG0baDcqqGObaAcqqGLbqzcqqGYbGCcqqG3bWDcqqGPbqAcqqGZbWCcqqGLbqzcqGGUaGlaaaacaGL7baaaaa@7EE0@

where *κ *∈ ℝ and *ρ*, the *pheromone evaporation rate*, are two tuning constants, *s*^*best *^is one of the tree s* or *s*_*k *_(see below), and lG|Γ|
 MathType@MTEF@5@5@+=feaafiart1ev1aaatCvAUfKttLearuWrP9MDH5MBPbIqV92AaeXatLxBI9gBaebbnrfifHhDYfgasaacPC6xNi=xH8viVGI8Gi=hEeeu0xXdbba9frFj0xb9qqpG0dXdb9aspeI8k8fiI+fsY=rqGqVepae9pg0db9vqaiVgFr0xfr=xfr=xc9adbaqaaeGacaGaaiaabeqaaeqabiWaaaGcbaGaemiBaW2aaSbaaSqaamrtHrhAL1wy0L2yHvtyaeHbnfgDOvwBHrxAJfwnaGabaiab=zq8hnaaBaaameaadaabdaqaaiabfo5ahbGaay5bSlaawIa7aaqabaaaleqaaaaa@3DA2@ the length of *s*^*best*^. When applying equation (8), if *τ*_*ij *_is greater than *τ*_*max *_or smaller than *τ*_*min*_, then its value is set to *τ*_*max *_or *τ*_*min*_, respectively. We set to *ρ *0.1, *κ *to *κρ *∈ [10^-2^, 10^-1^], and *α *to 0.7. Fine-tuning of these parameters might have a significant impact on search efficiency but such a systematic analysis is out of the scope of a proof-of concept for the use of ACO-ME. Finally, if the objective function does not decrease after 30 iteration, ACO-ME chooses *s*_*k *_as *s*^*best *^instead of s* for the pheromone updating; if the objective function does not decrease after 30 additional iterations, then all {*τ*_*ij*_} are reset to 0.5 and s* is used for pheromone updating.

### Parameter settings

We evaluated the performances of the ACO-ME algorithm under different values of the parameter *κ*(0.1, 0.5, and 1), and different numbers of ants (1 to 10). For each of the 30 possible combinations of these parameters values, we run ACO-ME for 1000 iterations. As suggested elsewhere (see [29]), we do not consider colony sizes larger than 10.

**Figure 4:**
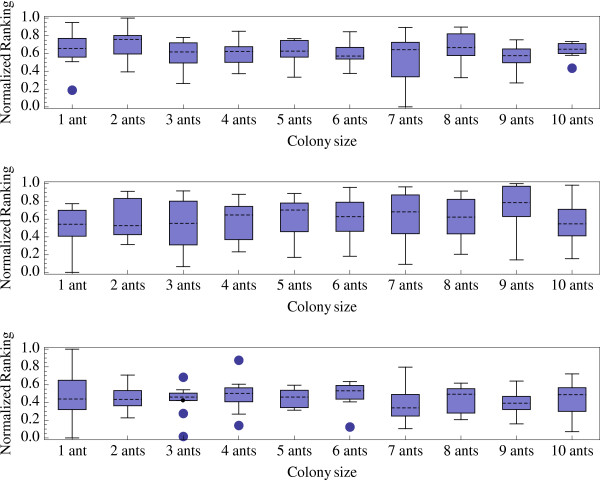
Normalized ranking of the ACO algorithm performances with small datasets (20 taxa) and *κ *= 0.1 (a), *κ *= 05 (b), and *κ *= 1 (c) versus colony size *n*_*a*_.

**Figure 5:**
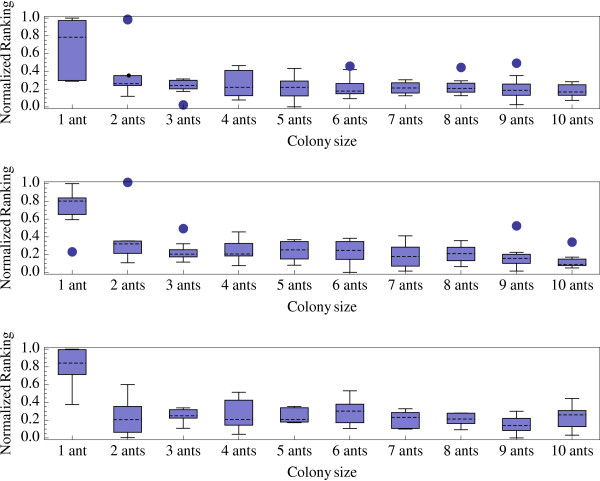
Normalized ranking of the ACO algorithm performances with medium datasets (50 taxa) and *κ *= 0.1 (a), *κ *= 0.5 (b), and *κ *= 1 (c) versus colony size *n*_*a*_.

**Figure 6:**
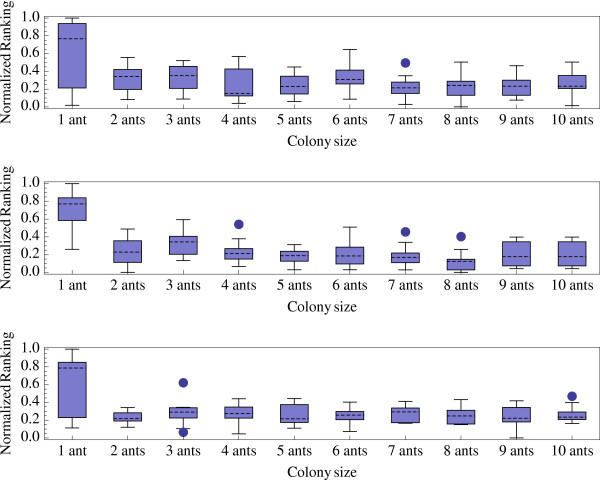
Normalized ranking of the ACO algorithm performances with large datasets (100 taxa) and *κ *= 0.1 (a), *κ *= 0.5 (b), and *κ *= 1 (c) versus colony size *n*_*a*_.

Relative performances are measured using a normalized index as in [39-41]:

Ijk=xj(x)−xjminxjmax−xjmin
 MathType@MTEF@5@5@+=feaafiart1ev1aaatCvAUfKttLearuWrP9MDH5MBPbIqV92AaeXatLxBI9gBaebbnrfifHhDYfgasaacPC6xNi=xI8qiVKYPFjYdHaVhbbf9v8qqaqFr0xc9vqFj0dXdbba91qpepeI8k8fiI+fsY=rqGqVepae9pg0db9vqaiVgFr0xfr=xfr=xc9adbaqaaeGacaGaaiaabeqaaeqabiWaaaGcbaqcfaOaemysaK0aa0baaeaacqWGQbGAaeaacqWGRbWAaaGaeyypa0ZaaSaaaeaacqWG4baEdaqhaaqaaiabdQgaQbqaaiabcIcaOiabdIha4jabcMcaPaaacqGHsislcqWG4baEdaqhaaqaaiabdQgaQbqaaiabd2gaTjabdMgaPjabd6gaUbaaaeaacqWG4baEdaqhaaqaaiabdQgaQbqaaiabd2gaTjabdggaHjabdIha4baacqGHsislcqWG4baEdaqhaaqaaiabdQgaQbqaaiabd2gaTjabdMgaPjabd6gaUbaaaaaaaa@4F10@

where xj(k)
 MathType@MTEF@5@5@+=feaafiart1ev1aaatCvAUfKttLearuWrP9MDH5MBPbIqV92AaeXatLxBI9gBaebbnrfifHhDYfgasaacPC6xNi=xH8viVGI8Gi=hEeeu0xXdbba9frFj0xb9qqpG0dXdb9aspeI8k8fiI+fsY=rqGqVepae9pg0db9vqaiVgFr0xfr=xfr=xc9adbaqaaeGacaGaaiaabeqaaeqabiWaaaGcbaGaemiEaG3aa0baaSqaaiabdQgaQbqaaiabcIcaOiabdUgaRjabcMcaPaaaaaa@31E7@ is the best solution found under parameter value *k *using dataset *j*, whereas xjmin
 MathType@MTEF@5@5@+=feaafiart1ev1aaatCvAUfKttLearuWrP9MDH5MBPbIqV92AaeXatLxBI9gBaebbnrfifHhDYfgasaacPC6xNi=xH8viVGI8Gi=hEeeu0xXdbba9frFj0xb9qqpG0dXdb9aspeI8k8fiI+fsY=rqGqVepae9pg0db9vqaiVgFr0xfr=xfr=xc9adbaqaaeGacaGaaiaabeqaaeqabiWaaaGcbaGaemiEaG3aa0baaSqaaiabdQgaQbqaaiabd2gaTjabdMgaPjabd6gaUbaaaaa@32F9@ and xjmax
 MathType@MTEF@5@5@+=feaafiart1ev1aaatCvAUfKttLearuWrP9MDH5MBPbIqV92AaeXatLxBI9gBaebbnrfifHhDYfgasaacPC6xNi=xH8viVGI8Gi=hEeeu0xXdbba9frFj0xb9qqpG0dXdb9aspeI8k8fiI+fsY=rqGqVepae9pg0db9vqaiVgFr0xfr=xfr=xc9adbaqaaeGacaGaaiaabeqaaeqabiWaaaGcbaGaemiEaG3aa0baaSqaaiabdQgaQbqaaiabd2gaTjabdggaHjabdIha4baaaaa@32FD@ are respectively the best and worst solutions found on the instance *j *using the parameter value *k*. By definition, performance index values are in the interval [0, 1]. The optimal parameter value exhibits the smallest relative performance index (see box-and-whisker plot histograms in Figure (4, 5, 6). Figures 4, 5, and 6 indicate that, for small, medium, and large datasets, the optimal combinations of number of ants/*κ *are 7/1, 10/0.5, and 8/0.5, respectively. However, differences of performances are not spectacular among different combinations of parameter values (except that performances are generally very low when a single at is used).

### Experimental evaluation

**Figure 7:**
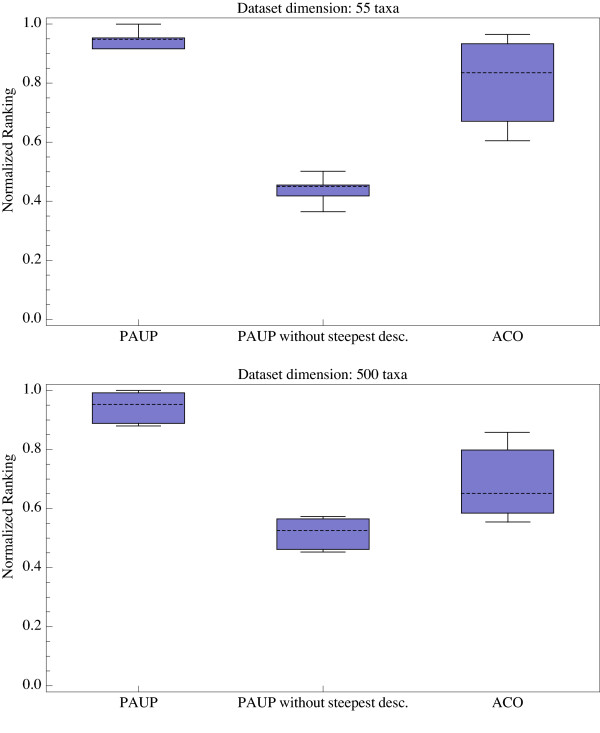
Comparison of performances between ACO-ME and hill-climbing (with and without Steepest Descent, SD) after a fixed run time of 1 minute on datasets of 55 (a) and 500 (b) taxa. A paired Wilcoxon test indicates that ACO-ME performances are significantly better (*p*-value = 3.92*e*^-2 ^for 55 taxa dataset, and *p*-value = 6.821*e*^-4 ^for 500 taxa dataset) than those of hill-climbing with SD, but significantly worst (*p*-value = 4.71*e*^-3 ^for 55 taxa dataset, and *p*-value = 4.53*e*^-4 ^for 500 taxa dataset) than those of hill-climbing without SD.

**Figure 8:**
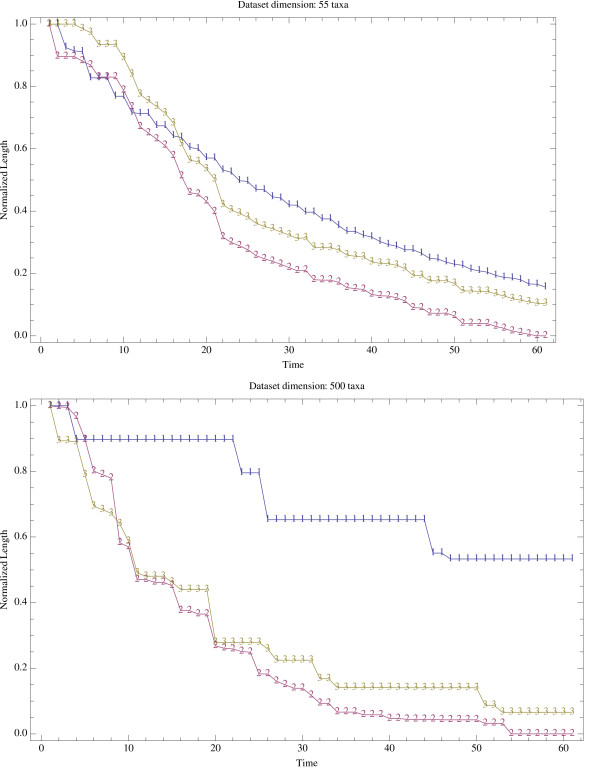
Comparison of score vs. running time for hill-climbing with steepest descent (line labeled "1"), hill-climbing without steepest decent (line labeled "2"), and ACO-ME (line labeled "3") on datasets of 55 (a) and 500 (b) taxa.

We first used a set of distance matrices generated from real datasets: the dataset "551314.nex" that includes 55 RBCL sequences of 1314 nucleotides each, and the dataset "Zilla500.nex" that includes 500 RBCL sequences of 1428 nucleotides each. These datasets are available at [42]. Note that sequences in these datasets were aligned using ClustalX [43] and columns including gaps were excluded before computing pairwise distances. Second, we generated (i) 10 artificial instances of 20 taxa (also called *small instances*); (ii) 10 artificial instances of 50 taxa (also called *medium instances*); and (iii) 10 artificial instances of 100 taxa (also called *large instances*). Each artificial instance was generated by random sampling of taxa and partial character reshuffling of the Zilla500.nex data set. More explicitly, after random selection of the 20 or 50 or 100 taxa, we randomly reshuffled characters among taxa, for 50 percents of the aligned columns. As the reshuffling makes the dataset prone to yield undefined pairwise distances [44], we simply used the absolute number of differences between sequence pairs for generating the distance matrix. Edge lengths were computed using the standard OLS because WLS and GLS can potentially lead to inconsistent results *et al*. [45]. All numerical experiments were performed on a workstation Apple 64-bit Power Mac G5 dual processor dual core, with 8 Gb of RAM, and OS X. The ACO-ME source code is written in C/C++ and compiled using IBM XL C/C++ compiler version 6.0. We compared the quality (total length) of trees generated by the ACO-ME algorithm to those obtained using a classical hill-climbing algorithm (implemented in PAUP* 4.0 [19]) after a fixed run time of 1 minute. The starting tree was generated using the Neighbor-Joining algorithm [20,21], and the TBR branch-swapping operator [6] was used for exploring the solution space. PAUP* 4.0 was used with and without the "Steepest Descent" (SD) option. When SD is activated, all possible TBR are tried, and the rearrangement producing the largest decrease in tree length is selected, inducing a computational overhead similar to that of the 2-OPT local search implemented in our ACO-ME algorithm. Each algorithm was run 30 times on each of the two real datasets. Figure 7a and 7b show that ACO-ME performances are intermediate between hill-climbing with SD, and hill-climbing without SD. Furthermore, Figure 7a and 7b indicate that the relative performances of ACO-ME, in comparison to hill climbing, increase with larger datasets. Note that, contrary to our simple implementation of ACO-ME, the implementation of ME in PAUP* 4.0 [19] incorporates procedures [4,23] that greatly speed-up the OLS (reaching a complexity *O*(*n*^2^)). We trust that implementation of these procedures in combination with further tuning of the ACO parameters (number of ants, relative weights of the heuristic information and stochastic pheromone parameters, etc) would lead to better performances of the ACO-ME algorithm. Figure 8a and 8b indicate that the relative performances described above are relatively stable trough time, especially for large data sets (at any time during the run, ACO-ME has similar performances than "hill-climbing without SD" and better performances than "hill-climbing with SD").

## Conclusion

We introduce here an Ant Colony Optimization algorithm (ACO) for the phylogeny estimation problem under the minimum evolution principle and demonstrate the feasibility of this approach. Although much improvement in performances can probably be obtained through (i) modification of the local search phase, (ii) tuning of the ACO parameters (number of ants, relative weights of the heuristic information and stochastic pheromone parameters, etc), and (iii) implementation of speed-up procedures and optimization of the code, the current implementation of our algorithm already demonstrates that the ant colony metaphor can efficiently solve instances of the phylogeny inference problem.

## Authors' contributions

All authors read and approved the final manuscript. Daniele Catanzaro, Raffaele Pesenti, and Michel C. Milinkovitch conceived the study and wrote the manuscript, Daniele Catanzaro performed the numerical analyses.

## Supplementary Material

Additional File 1 An ant colony optimization algorithm for phylogenetic estimation under the minimum evolution principle – supplementary material. The supplementary file includes discussions on the structure of the EPT matrices as well as how we generate and enumerate topologies in ACO-ME. Click here for file
